# Desmoplasia in pancreatic ductal adenocarcinoma: insight into pathological function and therapeutic potential

**DOI:** 10.18632/genesandcancer.171

**Published:** 2018-03

**Authors:** Andrew Cannon, Christopher Thompson, Bradley R. Hall, Maneesh Jain, Sushil Kumar, Surinder K. Batra

**Affiliations:** ^1^ Department of Biochemistry and Molecular Biology, University of Nebraska Medical Center, Omaha, NE, USA; ^2^ Department of Surgery, University of Nebraska Medical Center, Omaha, NE, USA; ^3^ Fred and Pamela Buffet Cancer Center, University of Nebraska Medical Center, Omaha, NE, USA

**Keywords:** pancreatic ductal adenocarcinoma, desmoplasia, cancer-associated fibroblast, extracellular matrix, SHH

## Abstract

Extensive desmoplasia is a prominent feature of the pancreatic ductal adenocarcinoma (PDAC) microenvironment. Initially, studies demonstrated that desmoplasia promotes proliferation, invasion and chemoresistance in PDAC cells. While these findings suggested the therapeutic potential of targeting desmoplasia in PDAC, more recent studies utilizing genetically-engineered mouse models of PDAC, which lack key components of desmoplasia, demonstrated accelerated progression of PDAC. This contrast calls into question the paradigm that desmoplasia unilaterally promotes PDAC progression and the premise of desmoplasia-targeted therapy. This review briefly examines the major reports of the tumor-promoting and -restraining roles of desmoplasia in PDAC with commentary on the gaps in our current understanding of desmoplasia in PDAC. Additionally, we discuss the studies demonstrating the heterogeneous and multifaceted nature of desmoplasia in PDAC and advocate for future areas of research to thoroughly address the various facets of desmoplasia in PDAC, reconcile seemingly contradictory reports of the role of desmoplasia in PDAC progression, and discover aspects of desmoplasia that are therapeutically actionable.

## INTRODUCTION

Pancreatic ductal adenocarcinoma (PDAC) is the third leading cause of cancer-related deaths in the United States [1]. The dismal prognosis of PDAC patients with PDAC is attributed to several factors including early disease dissemination due to which a majority of PDAC patients are diagnosed with the metastatic disease. As a result, few patients are eligible for curative surgical resection and the majority must be treated with systemic chemotherapy. Despite substantial progress in recent years, chemotherapy remains largely ineffectual in producing meaningful clinical responses or improving PDAC patient survival [2]. Several factors contribute to the failure of chemotherapy in PDAC including, the prevalence of desmoplasia in the PDAC tumor microenvironment (TME). Due to its ability to act as a biophysical barrier to therapeutic agents, TME is viewed as a promising target for augmenting the response of PDAC to therapeutic agents.

**Figure 1:**
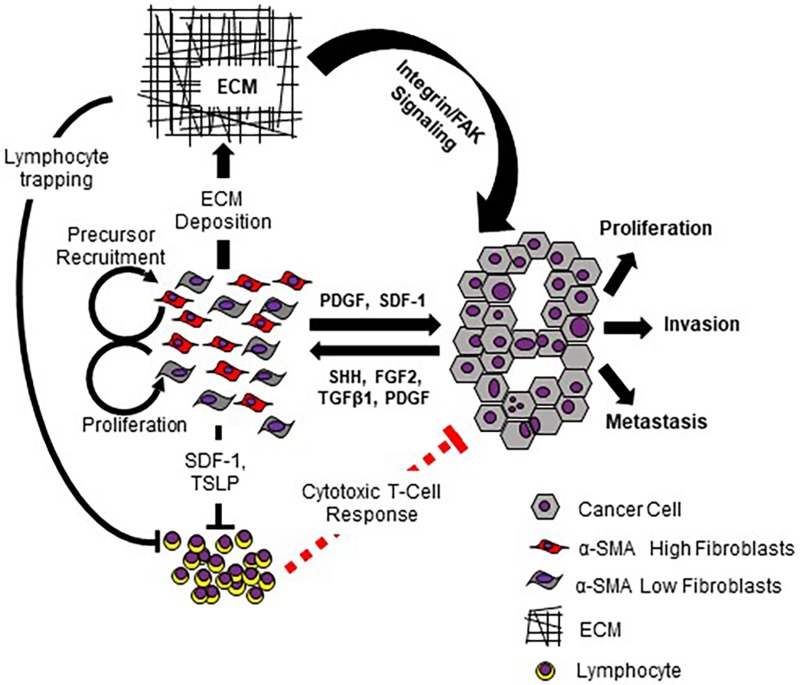
Schematic representation of the origin and role of the desmoplastic reaction in PDAC progression Cancer cells secrete multiple factors including SHH, FGF2, TGFβ1, and PDGF that result in CAF proliferation, recruitment of CAF precursors, and activation of CAFs. Activated CAFs, in turn, secrete factors that promote the proliferation, invasion, migration and metastatic features of PDAC cells. In addition, activated CAFs secrete immunosuppressive cytokines and components of extracellular matrix (ECM), obstructing tumor perfusion and developing the hypoxic environment. The dense ECM further contributes to PDAC progression through contact-mediated lymphocyte trapping and stimulation of Integrin/FAK signaling in PDAC cells.

Desmoplasia consisting largely of dense extracellular matrix (ECM) and fibroblasts is a prominent feature of the TME in PDAC. Initially, the role of desmoplasia in PDAC was overlooked. However, later studies showed that during the development of pancreatic cancer, cancer cells expend a great deal of energy to promote the recruitment, proliferation and activation of fibroblasts. Subsequently, activated fibroblast deposit extracellular matrix and secrete numerous factors that profoundly affect the behavior of cancer cells. Indeed, the preponderance of data from early studies demonstrated the tumor-promoting role of desmoplasia by driving tumor cell proliferation, promoting invasive properties and suppressing anti-tumor immune response. Furthermore, pharmacologic inhibition of desmoplasia in combination with chemotherapy slowed PDAC progression to a greater extent than PDAC alone, thereby highlighting desmoplasia as a potential therapeutic target in PDAC [3-6]. However, PDAC animal models lacking critical elements of the desmoplastic reaction used in recent studies exhibited accelerated PDAC progression. This contrast raises fundamental questions regarding the role of desmoplasia in PDAC progression and therapy resistance, and the basis of desmoplasia-targeted therapies (Figure 1).

This review analyzes the major studies investigating the significance of desmoplasia in PDAC. We accentuate both the contribution of each study to the understanding of desmoplasia as well as their limitations, which warrant caution in interpretation and generalization of the impact of desmoplasia in PDAC. From these analyses, we hope to gain insight into the mechanistic role of desmoplasia in PDAC, investigate the emerging concept of fibroblast heterogeneity in PDAC and comment on the future of desmoplasia-targeted therapies in PDAC.

### Desmoplasia as a facilitator of PDAC progression

Cancer-associated fibroblasts (CAFs) are a major constituent of the PDAC desmoplastic reaction and are responsible for most of the ECM deposition. Among various factors, PDAC cells secrete Transforming growth factor beta-1 (TGF-β1), Fibroblast growth factor-2 (FGF2), sonic hedgehog (SHH) and platelet-derived growth factor (PDGF) that drive fibroblast proliferation and ECM deposition, which in turn, profoundly affect the cancer cell behavior [7-10]. Subcutaneous injection of MiaPaCa-2, Panc-1 or SW850 PDAC lines with primary CAFs resulted in greater tumor volumes compared to tumors derived from cancer cells alone [10]. Similarly, mice orthotopically injected with primary CAFs and MiaPaCa-2 had larger tumors with dense fibrotic bands and increased α-SMA staining compared to mice injected with MiaPaCa-2 or CAFs alone [11]. Furthermore, tumors derived from co-implantation of cancer cells and fibroblasts had increased numbers of PCNA-positive cancer cells, suggesting that increased tumor volume was partly due to increased cancer cell proliferation. Finally, mice co-injected with cancer cells and CAFs exhibited increased loco-regional and distant metastasis. The *in vitro*, myofibroblast-conditioned media increased PDAC cell proliferation, migration, invasion, and decreased apoptosis. Additionally, CAFs increased expression of cancer stem cell and epithelial-mesenchymal transition markers in PDAC cells, further supporting the role of CAFs in metastasis and therapy resistance [12, 13].

CAFs have also been shown to promote immunosuppression in PDAC. CAF-derived thymic stromal lymphopoietin (TSLP) modulated dendritic cell (DC) cytokine profiles to favor Th2 polarization *in vitro.* Further analysis demonstrated that TSLP is derived from CAFs; correspondingly, TSLP receptor-positive DCs are found only in the tumor and tumor-draining lymph nodes [14]. Depletion of FAP-positive CAFs in an autochthonous mouse model decreased the growth of tumors and increased T-effector cell infiltration in a CXCL12/CXCR4 dependent manner [15]. Additional subsets of CAFs have been shown to promote differentiation of PBMCs to MDSCs *ex vivo* through STAT3 activation [16].

ECM deposition is another prominent feature of desmoplasia that is thought to promote PDAC progression. For instance, collagen I potentiates proliferation and chemoresistance of PDAC cells and limits T-cell accumulation near cancer cells [17, 18]. Further, ECM constituents increase intra-tumor interstitial fluid pressure (IFP) and matricellular tension (MCT) [19, 20]. Increased MCT in Kras^LSL-G12D^; TGFBR2^fl/fl^; Ptf1a-Cre (KTC) mice enhanced STAT3-mediated signaling in PDAC cells and accelerated disease progression compared to Kras^LSL-G12D^; TP53^R172H^; Pdx-1cre (KPC) mice [20]. Increased IFP also contributed to vascular dysfunction in KPC mice thereby limiting accumulation of chemotherapeutics within the tumor [19, 21] and increasing tumor hypoxia [21]. Hypoxia further modulates tumor-stroma crosstalk [22, 23], and selects more aggressive clones thereby contributing to the aggressiveness of the disease [24]. Cumulatively, these studies demonstrate that desmoplasia significantly contributes to rapid PDAC progression and are rationale for development of desmoplasia-targeted therapies for PDAC. However, studies utilizing genetically-engineered mouse models suggest a more nuanced role of desmoplasia in PDAC.

### Dichotomous role of desmoplasia in PDAC progression

#### Sonic Hedgehog (SHH) knockout (KO) mouse models of PDAC

SHH overexpression in PDAC cells promotes desmoplasia through paracrine signaling [9, 25, 26]. Several studies have examined the utility of targeting SHH pathway to reduce desmoplasia and improve the delivery of chemotherapeutic agents [9, 27]. However, inhibition of SHH for short term using pharmacological agents or its ablation at genetic level have suggested a dichotomous role of this pathway in PDAC pathobiology [27-29]. Two recent studies examined the impact of SHH pathway on PDAC desmoplasia and progression using SHH KO murine models. Rhim et al. generated SKPC (Pdx1-Cre;SHH^fl/fl^;Kras^LSL-G12D/+^;P53^fl/+^;Rosa26^LSL-YFP/+^) mice to deplete SHH expression in pancreatic epithelium. While SKPC pancreata lacked SHH, Indian Hedgehog (IHH) expression was increased. Despite this compensation, expression of Gli-1 in F4/80+ cells and pancreatic tumor decreased more than ten-fold indicating decreased HH signaling [29]. SKPC tumors showed decreased α-SMA staining and increased vascular density (CD31+ area) compared to KPC mice. Furthermore, SKPC mice developed undifferentiated tumors with increased expression of EMT markers [29]. Unexpectedly, SKPC mice had decreased survival and increased metastasis. Long-term inhibition of Smoothened with IPI-926 recapitulated SHH KO, and concomitant administration of gemcitabine did not rescue decreased survival indicating that increased gemcitabine delivery did not overcome the aggressive behavior of SKPC tumors. Interestingly, loss of SHH signaling sensitized tumors to VEGFR2 blockade suggesting that the aggressive behavior of SKPC tumors is mediated by increased vascular density [29].

Similarly, Lee et al. generated SKC (SHH^fl/fl^; Ptf1a-Cre; KrasLSL^−G12D^) and SKPC (SHH^fl/fl^; Ptf1a-CRE; KrasLSL^−G12D^; P53fl^/+^) murine PDAC models [28]. SKC mice showed robust, early development of PanIN lesions and increased propensity for development of PDAC by 55 weeks of age compared to Kras^LSLG12D^; Pdx-1cre (KC) animals. Further, SKC mice demonstrated persistence of PanIN lesions following cerulein-induced injury to pancreatic parenchyma. Consistent with Rhim et al., SKPC mice showed reduced survival and increased vascular density compared to KPC mice. Pharmacological modulation of SHH signaling with cerulein-accelerated carcinogenesis demonstrated an inverse relationship between hedgehog signaling and presence of PDX-1-positive EPCAM-positive progenitor cells suggesting that SHH signaling may constrain the expansion of pancreatic progenitor cells following inflammation [28].

Collectively, these data suggest several mechanisms through which SHH deletion may contribute to PDAC progression. First, changes in differentiation status, expression of EMT markers, and the persistence of PanIN lesion are plausibly due to disruption of SHH signaling that impairs production of CAF-derived factors required for suppression of observed neoplastic cell phenotypes. Second, lack of SHH driven ECM deposition may allow increased dissemination of cancer cells from the primary site to distant sites. As in the case of enzymatic depletion of ECM [19, 21], both Rhim and Lee also observed increased vascular density with loss of SHH. While increased vascular density and tumor perfusion increase the delivery of therapeutics to the tumor [21, 27, 29], they may also increase the opportunity for cancer cells to metastasize and alleviate stress associated with hypoxia or nutrient deprivation. Thus, the aggressive disease observed in these models is plausibly attributable to the overall loss of SHH-driven desmoplasia.

However, it remains difficult to discern the actual contribution of the loss of desmoplasia to the aggressive phenotype observed in SHH KO mice. Despite, studies demonstrating SHH's function in pancreatic mesenchyme [9, 26, 30], Lee et al. suggested that SHH may be critical for regeneration of exocrine pancreas and resolution of inflammation [28]. This is consistent with observations of prolonged pancreatic inflammation and persistence of inflammation and PanIN lesions in response to cearulein treatment and induction of Kras^−G12D^ in Gli1^fl/+^ animals [31]. Additional studies showed changes in gene expression in metaplastic pancreatic epithelium consistent with HH pathway activation [32, 33]. Most convincingly, Fendrich et al. showed that smoothened^fl/fl^ Pdx-Cre and Ela-Cre mice exhibited persistent acinar-to-ductal metaplasia seven days after cerulein treatment compared to wild-type mice, which showed complete recovery, suggesting that loss of HH signaling in pancreatic epithelium impairs the resolution of pancreatic inflammation [34]. Therefore, the aggressive phenotype of neoplastic cells following SHH deletion may in part be attributed to the loss of autocrine, SHH signaling.

Similarly, SHH deletion increased intratumoral vascular density, which was not the result of loss of SHH signaling in endothelium [29]. These findings are confirmed in a subcutaneous PDAC model in which PDAC lines were co-injected with WT fibroblasts or fibroblast with homozygous deletion of SHH co-receptors (GAS1^−/−^ and BOC^−/−^ or GAS1^−/−^, BOC^−/−^ and CDON^−/−^) [35]. Here, suppression of SHH signaling in fibroblasts (GAS1^−/−^, BOC^−/−^) increased angiogenesis through upregulation of fibroblast-derived angiopoietin-1 and 2 and augmented tumor growth. However, complete loss of SHH signaling in fibroblasts (GAS1^−/−^, BOC^−/−^ and CDON^−/−^) abrogated angiogenesis and tumor growth. Interestingly, suppression of SHH signaling in fibroblasts did not decrease desmoplasia or alter PDAC cell phenotype, which is consistent with findings from biopsies of a subset of patients treated with smoothened inhibitor, GDC-0449 [35, 36]. These observations suggest that compensatory upregulation of IHH in SKPC mice may contribute to increased vascular density and the aggressive disease observed in SKPC mice. Thus, increased vascular density rather than depletion of stroma *per se* may mediate aggressive disease course in SKPC mice [35]. It is, however, difficult to understand the impact of SHH co-receptor deletion on the course of the disease given that tumors in SKPC mice were smaller whereas GAS1^−/−^, BOC^−/−^ tumors were larger compared to respective controls. Regardless, these findings are, to some extent, mirrored in an Ela-myc model of PDAC, in which deletion of Galectin-1, a promoter of SHH signaling in PDAC, resulted in decreased angiogenesis, desmoplasia, and prolonged survival of mice [37]. Finally, Rhim et al., reported significant weight loss, reminiscent of cachexia, in SKPC mice. While the mechanism of the observed weight loss is unknown, it represents another aspect of the SKPC phenotype that confounds interpretation of the role of stromal depletion in PDAC progression [29].

Importantly, both SHH^−/−^ PDAC models replicate the findings from clinical trials of smoothened inhibitors in PDAC. Phase I trials of IPI-926 showed potential, but a phase II trial was halted due to decreased survival in patients receiving IPI-926 [38]. Similarly, another smoothened inhibitor, GDC-0449 in phase I and II clinical trials with gemcitabine failed to demonstrate benefit over gemcitabine, as well as compared to historical and placebo controls [36, 39]. These trials demonstrated that SHH blockade reduced desmoplasia and increased tumor perfusion in a subset of patients, but these changes did not correlate with patient survival [36, 39]. Rhim et al. and Lee et al. suggest that this lack of efficacy may result from the emergence of more aggressive disease with the loss of SHH. Overall, SHH^−/−^ models helped elucidate the changes in the TME due to loss of SHH signaling and unraveled mechanisms that contributed to the failure of SHH inhibitors in clinical trials. However due to the pleiotropic effects of SHH deletion, it is challenging to tease out the intricacies of the interplay between SHH and desmoplasia in PDAC initiation and progression.

### α-SMA; Thymidine Kinase model of PDAC

**Figure 2:**
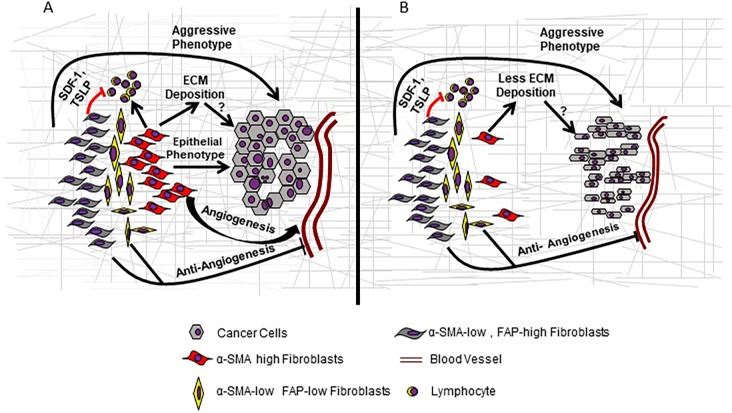
Model of CAF heterogeneity in PDAC **A.** Based on the phenotypic characteristics, inflammatory (α-SMA low, FAP high) and myofibroblast (α-SMA high, FAP low) CAF subsets exist in a dynamic equilibrium during tumor progression. The crosstalk and secretome of the heterogeneous CAF populations create a unique microenvironment affecting infiltrating immune cells, tumor vasculature and cancer cells that dictate their dichotomous role during early and late phases of tumor development. **B.** Depletion of the myofibroblasts subset allows the predominance of inflammatory CAFs in the TME leading to suppression of anti-tumor immune response, reduced ECM deposition and angiogenesis as well as a poorly differentiated cancer cell phenotype.

The α-SMA-positive CAFs (pancreatic stellate cells-PSCs PSCs / myofibroblasts) are an abundant population of CAFs in PDAC. To determine the contribution of α-SMA-positive CAFs to PDAC progression, Ozdemir et al. cloned human herpes virus thymidine kinase under the α-SMA promoter into Ptf1a-Cre;Kras^LSL-G12D^;Tgfbr2^fl/fl^ mice thereby sensitizing α-SMA-positive cells to ganciclovir and allowing temporal control of myofibroblast depletion [40]. Mice treated with ganciclovir demonstrated modest reduction of desmoplasia with a marked decrease in α-SMA-positive cells. As in previous studies, myofibroblasts-depleted tumors exhibited less differentiated histology with increased expression of EMT markers and CD133+ cells (PDAC stem cell marker), and reduced survival for early and late depletion groups, which was not rescued by administration of gemcitabine. Ganciclovir-treated mice experienced marked weight loss, as observed by Rhim et al. and exhibited increased incidence of pulmonary embolism. Despite this similarity, myofibroblasts-depleted tumors showed decreased tumor vasculature implying that increased vascularity in SHH KO studies may be specific to suppression of SHH signaling. Interestingly, gene expression analysis suggested that myofibroblast depletion caused significant alterations in the tumor immune environment. Analysis of intratumoral lymphoid populations revealed decreased CD4+ T-eff and CD8+ to CD4+, Foxp3+ ratios along with increased expression of Ctla-4 while changes in myeloid populations showed decreased numbers of macrophages and an increased number of granulocytes. Administration of Ctla-4 blocking antibody reversed changes in immune infiltrate as well as tumor vasculature and histology suggesting that observed changes may be rooted in altered tumor immune response following myofibroblast depletion (Figure 2). Following this observation, the same group published a report of multiplexed T-cell staining demonstrating that in human samples α-SMA and collagen I staining near tumor cells does not correlate with lack of T-cells in the same region further supporting these claims. Subsequently, using multiplexed T-cell staining, it was demonstrated that there was no correlation between alpha-SMA and collagen staining around tumor cells and the absence of T-cells in the same regions of the tumor [41]. In contrast to these findings, Jiang et al. demonstrated that knockdown of focal adhesion kinase (FAK) signaling in PDAC cells abrogated FAP-positive CAFs and collagen I deposition and allowed for T-cell-dependent inhibition of tumor progression and increased survival of mice [42]. Similar to the study by Rhim et al. it is difficult to infer from these studies if stromal depletion was causative of increased T-eff cell infiltration and tumor inhibition because FAK invariably has multiple other effects on the PDAC TME. Specific depletion of α-SMA-positive cells by Ozdemir et al. provides significant insight into the role of myofibroblasts in PDAC progression. Importantly, the loss of α-SMA-positive fibroblasts from the TME results in changes in both tumor histology and immune cell infiltrate. The similarity of changes in histology between this study and that of Rhim et al. suggests that α-SMA positive fibroblasts may directly modulate the differentiation status and EMT of PDAC cells. However, the contribution of these changes to PDAC progression after stromal depletion is unclear as neither study demonstrates that these features are causative of poor survival. While the specific depletion of α-SMA positive cells by Ozdemir et al. led to the better understanding of the contribution of myofibroblasts, these studies do not provide any information regarding the role of other fibroblast subtypes, like FAP-positive cells, which have emerged as a major immunosuppressive population in PDAC [15]. Additionally, recent evidence shows that pro-tumorigenic, cytokine-secreting fibroblast populations lose expression of α-SMA and may not be depleted in this model [43].

As in experimental models, correlation of desmoplasia with clinical outcomes show conflicting results. Using gene expression analysis from 145 primary and 61 metastatic tumors, Moffit et al. identified normal and activated stroma gene signatures. Patients with activated stroma signatures had worse survival compared to patients with similar tumor types and normal stroma signatures [44]. Another study showed that IHC analysis of Collagen I and Hyaluronan in primary tumors correlated with poor survival (*n* = 53) [45]. In contrast, radiographic assessment of apparent diffusion coefficient (ADC) correlated inversely with epithelial cellularity and positively with stromal content assessed by Movat's staining, which is consistent with findings in KPTC (Kras^LSLG12D/WT^;Ptf1a-CRE;Tp53^fl/WT^;Ela-Tgfa) and other mouse models [29, 40, 46]. Importantly, ADC correlated with longer patient survival in 96 early stage patients [46].

## CONCLUSION AND PERSPECTIVES

An important consequence of these three major studies which failed to support the pro-tumorigenic contribution of the desmoplastic reaction is the appreciation of fibroblast heterogeneity in the PDAC TME. While there is much speculation on the subject, only one study has demonstrated clear differences in the activity of fibroblasts based on the expression of FAP and α-SMA [47]. Here, it was shown that α-SMA fibroblasts are most prevalent in close proximity to cancer cells both in murine tumor tissue as well as in organoid culture. In contrast, fibroblasts located further from cancer cells demonstrated decreased α-SMA expression and increased FAP expression. These two populations of cells were found to be mutually exclusive and have markedly different activities in the models tested. Importantly, analysis of these two cell populations showed that FAP-positive fibroblasts secrete a variety of cytokines including IL-6 which was shown to profoundly increase the longevity of fibroblasts grown in culture [47] and has been shown in several other studies to promote the malignant behavior of cancer cells [48]. These observations support the existence of multiple subtypes of fibroblasts with markedly different activities and presumably different effects on the progression of PDAC leading to both tumor-promoting and restraining roles in PDAC. With respect to this, the findings by Ohlund et al. suggest that depletion of the FAP-positive fibroblasts rather than the desmoplastic reaction as a whole is a promising avenue for pursuing desmoplasia-targeted therapy in PDAC. The field of fibroblast heterogeneity is still in its infancy, and more subpopulations of fibroblasts and sub-classifications within the existing classes will undoubtedly be identified. It will be important to determine the dynamics, plasticity and origins of these heterogeneous population during the evolution of PDAC from preneoplastic lesions to metastatic disease.

Ultimately, it is too early to accurately discern the role of desmoplasia as a whole in PDAC progression. Moving forward, an improved understanding of fibroblast biology including the cellular origins of fibroblasts in PDAC and the transcription factor networks driving fibroblast phenotypes both in physiological and pathological conditions will be critical. This understanding can be leveraged for the generation of murine models to specifically identify and characterize heterogeneous CAF populations present in the PDAC TME. Additionally, such insight will allow the generation of mouse models with conditional deletion of ECM components to begin to address the role of ECM in PDAC progression *in vivo*. With such studies, a clearer picture of the complexity of the PDAC desmoplastic reaction will emerge along with an understanding of the duality of desmoplasia and its role in PDAC progression.

Finally, while various mouse models have suggested that desmoplasia may play a tumor-restraining role in PDAC, the potential of desmoplasia as a therapeutic target has not diminished. Recent work demonstrating the presence of multiple CAF populations indicates that in the PDAC TME pro-tumorigenic and tumor-restraining components may coexist. Therefore, the discovery and characterization of distinct populations of CAFs may yield promising new targets for therapeutic intervention. Similarly, the activities of CAFs in PDAC have yet to be fully characterized. As with targeted therapy against cancer cells, it may not be advisable to deplete an entire fibroblast population from a tumor, but rather target a specific subset or activity of these cells to undermine the tumor supporting role or augment the tumor-restraining activities. The direct modulation of the extracellular matrix properties using a recombinant PEGylated hyaluronidase showed promising results in phase II clinical trials and is now in phase III testing [49]. While the mechanistic basis of the efficacy of hyaluronidase has yet to be fully elucidated, the findings from clinical trials suggest that the ECM may be a valid target for drug discovery. Overall, desmoplasia represents a key facet of PDAC biology, and while the precise role of desmoplasia in PDAC progression remains elusive, there are numerous aspects of desmoplasia that continue to have potential as therapeutic targets for the treatment of PDAC.
